# Real‐world treatment patterns and outcomes in accelerated and blast‐phase myeloproliferative neoplasms: Insights from a large multi‐centre cohort analysis in the United Kingdom

**DOI:** 10.1111/bjh.70511

**Published:** 2026-05-03

**Authors:** Alexandros Rampotas, Gabriel Naylor‐Layland, Clare Brown, Ahmad Alabdulkarim, Jennifer Ryan, Frances Wadelin, Samah Alimam, Phyo Wint Wint Tun, Jonathan Lambert, Jennifer O'Sullivan, Andrew J. Wilson, Laith Tafesh, Andrew McGregor, Simone Claudiani, Andrew Innes, Emily Booth, James Leveson, Steve Knapper, Mamta Garg, Mani Dubey, Theodora Vatopoulou, Charlotte Brierley, Wai Ka Natalie Leung, Graham Greenfield, Mary Frances McMullin, Alesia Khan, Kate Milne, Duncan Brian, Anna Godfrey, Claire N. Harrison, Bethan Psaila, Patrick Harrington, Amy Kirkwood, Tim C. P. Somervaille, Donal P. McLornan, the UK Blood Cancer Research Network MPN Clinical Study Group

**Affiliations:** ^1^ University College London Hospitals NHS Foundation Trust London UK; ^2^ CRUK Cancer Trials Centre, UCL London UK; ^3^ The Christie NHS Foundation Trust London UK; ^4^ Cancer Research UK Manchester Institute Manchester UK; ^5^ Nottingham University Hospitals NHS Foundation Trust Nottingham UK; ^6^ Guy's and St Thomas' NHS Foundation Trust London UK; ^7^ The Newcastle upon Tyne NHS Foundation Trust Newcastle upon Tyne UK; ^8^ Imperial College Healthcare NHS Trust London UK; ^9^ University Hospital of Wales Cardiff UK; ^10^ School of Medicine Cardiff University Cardiff UK; ^11^ University Hospitals of Leicester NHS Trust Leicester UK; ^12^ St George's NHS Foundation Trust London UK; ^13^ Oxford University Hospitals NHS Foundation Trust Oxford UK; ^14^ Belfast Health and Social Care Trust Belfast UK; ^15^ Leeds Teaching Hospitals NHS Foundation Trust Leeds UK; ^16^ Cambridge University Hospitals NHS Foundation Trust Cambridge UK

**Keywords:** accelerated phase, blast phase, MPN, myeoproliferative neoplasms, real world

## Abstract

This UK‐based retrospective analysis describes real‐world treatment patterns and outcomes in 175 patients with accelerated (AP, *n* = 69) or blast‐phase (BP, *n* = 106) ‘Philadelphia‐negative’ myeloproliferative neoplasms (MPN‐AP/BP) diagnosed between 2013 and 2025. Median age at transformation was 71 years. With a median follow‐up of 45.2 months, median overall survival (OS) was 14.9 months, significantly worse for MPN‐BP (6.7 months) versus MPN‐AP (25.3 months). Treatment selection was heterogeneous across centres. Intensive chemotherapy (IC) improved outcomes only when followed by allogeneic haematopoietic stem cell transplant (allo‐HSCT) (median OS 24.7 months). Ruxolitinib‐based regimens, particularly combined with azacitidine, showed acceptable activity in AP (median OS 27.2 months). Venetoclax‐based regimens achieved a median OS of 14.9 months across the cohort. Multivariable analysis identified IC and venetoclax‐based therapy as independently associated with better outcomes, reflecting patient selection, while *TP53* mutations predicted inferior survival. IC carried high rates of febrile neutropenia and sepsis; venetoclax was associated with prolonged cytopenias. This study confirms the poor prognosis of MPN‐AP/BP, the absence of a unified UK consensus approach and the need for improved therapies and prospective studies to determine optimal treatment approaches for this challenging cohort.

## INTRODUCTION

Classical ‘Philadelphia‐negative’ myeloproliferative neoplasms (MPNs) are a heterogeneous group of clonal haematopoietic stem cell disorders encompassing polycythaemia vera (PV), essential thrombocythaemia (ET) and myelofibrosis (MF). These disorders are characterised by constitutive activation of the JAK/STAT signalling pathway,^1^ most commonly driven by somatic ‘driver’ mutations in *JAK2*, *CALR* or *MPL*.^2^, ^3^, ^4^ Despite a generally chronic clinical course in many patients, all MPN subtypes carry an inherent yet variable risk of disease progression to an accelerated (MPN‐AP) or blast‐phase (MPN‐BP) myeloproliferative neoplasm. Conventionally, these transformation stages are defined by the percentage of blasts in the peripheral blood or bone marrow, MPN‐AP having 10%–19% blasts and MPN‐BP ≥20% blasts.^5^ Cumulative incidences of transformation to MPN AP/BP varies among MPN subtypes, affecting approximately 2%–4% of patients with ET, 3%–6% with PV and up to 10%–20% with primary MF.^6^, ^7^ In all cases, such transformation frequently associates with a markedly adverse prognosis. Estimated median overall survival (OS) for patients with MPN‐AP is typically 12–18 months, at best, whereas that of MPN‐BP remains dismal at 3–6 months for the majority, even with intensive therapy,^8^ while there is lack of prospective clinical studies available to inform best treatment strategies. The only potentially curative approach remains an allogeneic haematopoietic stem cell transplant (allo‐HSCT).

Blastic transformation of MPNs is commonly driven by the acquisition of additional somatic mutations and cytogenetic abnormalities that confer clonal evolution and therapy resistance.^9^ So‐called ‘high‐risk’ mutations frequently observed at the time of transformation include pathogenetic mutations in *TP53, ASXL1, RUNX1, SRSF2* and *IDH*1/2, while complex or monosomal karyotypes are common and are associated with particularly adverse outcomes.^10^, ^11^ Distinct biological routes to transformation have been proposed: one involving stepwise acquisition of secondary mutations within the *JAK2/CALR/MPL*‐mutated clone, often in association with TP53 disruption,^12^ and another involving emergence of an independent leukaemic clone, frequently associated with loss or reduction of the ‘founding driver’ mutation. More recently, chromothripsis has been recognised as a landmark feature that is associated with progression to MPN‐AP/BP disease.^13^ Chromothripsis leads to recurrent amplification of chromosome 21, with *DYRK1A* overexpression and a resultant more ‘open’ chromatin which induces increased proliferation and progression of the disease towards a blast‐phase/leukaemic phenotype.

Therapeutic advances in acute myeloid leukaemia (AML) over the last decade, including the introduction of venetoclax‐based regimens and IDH inhibitors, have been extrapolated to use in MPN‐AP/BP despite their distinct biology, alongside conventional intensive chemotherapy (IC) and combinations of hypomethylating agents (HMAs) with JAK inhibitors. However, clinical outcomes, in general, remain poor. Real‐world studies report median OS estimates of 4–8 months for venetoclax‐based regimens, 10–15 months with IDH inhibitors and 7–9.5 months with HMAs plus ruxolitinib combinations.^14^, ^15^, ^16^, ^17^ Responses remain variable and refractory disease is common.

Allo‐HSCT remains the only potentially curative option, though its applicability is frequently limited by advanced patient age, co‐morbidities and high treatment‐related morbidity and mortality.^18^ Given the limited prospective data and global heterogeneity in treatment approaches, optimal management of MPN‐AP/BP remains undefined. Furthermore, assessment of therapeutic response has varied across studies, with both AML‐specific and MPN‐adapted criteria applied inconsistently. To address these gaps, we conducted a large multicentre retrospective analysis of adults diagnosed with MPN‐AP/BP across large UK centres since 2013. We aimed to evaluate survival outcomes according to treatment strategy, assess the prognostic impact of baseline characteristics and examine outcomes following allo‐HSCT in this population.

## METHODS

We conducted a retrospective, multicentre cohort study of patients with MPN who transformed to MPN‐AP or ‐BP between 18 February 2013 and 21 February 2025. MPN‐AP and ‐BP were defined according to the WHO handbook, 6th Edition. Cases were identified through institutional databases. Clinical, molecular and treatment specific data were abstracted from medical records. Among 192 patients identified, 17 were excluded because of incomplete diagnostic data, leaving a total of 175 evaluable patients (MPN‐AP (*n* = 69) and MPN‐BP (*n* = 106)), who met definitions based on sustained blast% in the peripheral blood/bone marrow. The study was conducted in accordance with the Declaration of Helsinki and approved by institutional review boards of participating centres. Driver mutation (*JAK2*, *CALR* and *MPL*) and non‐driver mutation status (*ASXL1, TP53, TET2, DNMT3A, EZH2, CBL, U2AF1, SRSF2, IDH1/2, KRAS* and *NRAS*) were recorded from next‐generation sequencing or targeted molecular assays performed at the time of transformation. Patients lacking *JAK2, CALR* and *MPL* mutations were designated as ‘triple negative’ MPN (*n* = 14, 8.5%). Patients were categorised by initial therapy employed following transformation into six groups (Table S1): Azacitidine monotherapy, IC, venetoclax‐based therapy, ruxolitinib, subdivided as monotherapy or combined with azacitidine, other therapies (including low‐dose cytarabine (*n* = 3), hydroxycarbamide (*n* = 2) and investigational agents (*n* = 3)) and no treatment. Allo‐HSCT was recorded as a specific cohort.

OS was defined from the date of MPN‐AP/BP diagnosis to death from any cause or last follow‐up. Progression‐free survival (PFS) was defined from diagnosis to relapse, progression or death. Patients proceeding to allo‐HSCT were censored at the time of transplant in sensitivity analyses. Clinical responses were investigator assessed according to revised response criteria for myelofibrosis: International Working Group‐Myeloproliferative Neoplasms Research and Treatment (IWG‐MRT) and European LeukemiaNet (ELN).

Categorical variables were compared using Pearson's chi‐squared test. OS and PFS were estimated by Kaplan–Meier methods and compared using log‐rank tests. Hazard ratios (HRs) were derived from Cox proportional hazards models; proportional hazard assumptions were tested and violations noted. Predictors of favourable outcome—defined as survival >1 year and/or complete or partial remission—were assessed using univariable logistic regression. Multivariable logistic regression including age, sex, treatment group, molecular profile and prior therapy was also used to predict favourable outcome which was defined as survival of >1 year, proceeding to an allo‐HSCT or achieving PR or better. Analyses were performed using Stata version 18.

## RESULTS

Patient and disease characteristics are summarised in Table 1. Median age at transformation was 71 years (interquartile range [IQR], 62–76); 110 of 175 patients (63%) were male. Of 82 patients with primary MF, the median time to transformation was 3.7 years (IQR, 1.4–7.7). For those with ET/PV, median time to transformation was 9.9 years (IQR, 4.7–15.4) when they progressed directly to AP/BP and 13.1 years (IQR 5.7–18.9) when diagnosed with post‐ET/PV MF prior to transformation respectively. Regarding driver mutation status, *JAK2* mutation was the most common in both MPN‐AP (69.5%) and MPN‐BP (52.2%), with *CALR* mutations (either type 1 or type 2) detected in 26.1% and 19.2% of MPN‐AP and MPN‐BP patients respectively. At time of transformation, where molecular annotation was available, the most frequent additional detected mutations were *ASXL1* (16%) and *TET2* (16%) across all cases, with pathogenetic mutations in *TP53* detected in 21.7% and 10.1% of MPN‐BP and MPN‐AP cases respectively.

**TABLE 1:** Baseline characteristics.

	All	AP	BP	*p*‐value*
	*N* = 175	*N* = 69	*N* = 106	
Age (years), median (IQR)	71.0 (62.0–76.0)	70.0 (64.0–75.0)	71.5 (62.0–77.0)	0.68
Range	17–88	36–86	17–88	
Sex, *N* (%)				0.85
Female	63 (36.4)	25 (37.3)	38 (35.8)	
Male	110 (63.6)	42 (62.7)	68 (64.2)	
Missing/unknown	2	2	0	
Prior diagnosis, *N* (%)				<0.001
Primary MF	57 (32.9)	36 (52.2)	21 (20.2)	
Secondary MF	42 (24.3)	15 (21.7)	27 (26.0)	
PV/ET	74 (42.8)	18 (26.1)	56 (53.8)	
Missing/unknown	2	0	2	
DIPSS+ score within 3 months of diagnosis, *N* (%)				<0.001
High	33 (26.4)	19 (38.0)	14 (18.7)	
Int‐1	11 (8.8)	4 (8.0)	7 (9.3)	
Int‐2	30 (24.0)	18 (36.0)	12 (16.0)	
Low	4 (3.2)	1 (2.0)	3 (4.0)	
Not applicable	47 (37.6)	8 (16.0)	39 (52.0)	
Missing/unknown	50	19	31	
Mutations				
Driver, *N* (%)				0.16
*JAK2*	109 (66.1)	36 (57.1)	73 (71.6)	
*Calreticulin*	38 (23.0)	18 (28.6)	20 (19.6)	
*MPL W515L*	4 (2.4)	3 (4.8)	1 (1.0)	
Triple negative	14 (8.5)	6 (9.5)	8 (7.8)	
Missing/unknown	10	6	4	
Secondary mutations, *N* (%)				
*ASXL1*	28 (16.0)	12 (17.4)	16 (15.1)	0.69
*DNMT3*	13 (7.4)	7 (10.1)	6 (5.7)	0.27
*TET2*	28 (16.0)	13 (18.8)	15 (14.2)	0.41
*EZH2*	10 (5.7)	5 (7.2)	5 (4.7)	0.52
*CBL*	3 (1.7)	2 (2.9)	1 (0.9)	0.56
*U2AF1*	5 (2.9)	3 (4.3)	2 (1.9)	0.38
*SRSF2*	17 (9.7)	7 (10.1)	10 (9.4)	0.88
*IDH1*	6 (3.4)	1 (1.4)	5 (4.7)	0.41
*IDH2*	10 (5.7)	5 (7.2)	5 (4.7)	0.52
*NRAS*	6 (3.4)	4 (5.8)	2 (1.9)	0.21
*KRAS*	3 (1.7)	2 (2.9)	1 (0.9)	0.56
*TP53*	30 (17.1)	7 (10.1)	23 (21.7)	0.05
No other mutation	39 (22.3)	16 (23.2)	23 (21.7)	0.82
Mutation not listed	92 (52.6)	35 (50.7)	57 (53.8)	0.69
High‐risk mutations (MIPSS70+), *N* (%)				0.09
At least 1	53 (30.3)	26 (37.7)	27 (25.5)	
None	122 (69.7)	43 (62.3)	79 (74.5)	
Treatments				
No. prior treatments, *N* (%)				0.14
0	13 (8.0)	6 (9.1)	7 (7.2)	
1	80 (49.1)	27 (40.9)	53 (54.6)	
2	42 (25.8)	17 (25.8)	25 (25.8)	
3	20 (12.3)	12 (18.2)	8 (8.2)	
4	6 (3.7)	3 (4.5)	3 (3.1)	
5	2 (1.2)	1 (1.5)	1 (1.0)	
Missing/unknown	12	3	9	
Prior treatments, *N* (%)				
Ruxolitinib	62 (42.8)	35 (62.5)	27 (30.3)	<0.001
Fedratinib	6 (4.4)	2 (4.0)	4 (4.7)	0.61
Momelotinib	4 (3.0)	3 (6.0)	1 (1.2)	0.14
Interferon A	17 (12.1)	5 (9.4)	12 (13.8)	0.6
Hydroxycarbamide	84 (48.0)	26 (37.7)	58 (54.7)	0.03
Allo‐HSCT	8 (4.6)	2 (2.9)	6 (5.7)	0.48
Treatment at AP/BP diagnosis, *N* (%)				
Ruxolitinib	53 (30.3)	29 (42.0)	24 (22.6)	0.01
Fedratinib	5 (2.9)	2 (2.9)	3 (2.8)	0.98
Momelotinib	3 (1.7)	3 (4.3)	0	0.06
Interferon A	6 (3.4)	0	6 (5.7)	0.08
Hydroxycarbamide	68 (38.9)	17 (24.6)	51 (48.1)	0.002
Azacitidine	8 (4.6)	4 (5.8)	4 (3.8)	0.71
Erythropoietin	10 (5.7)	8 (11.6)	2 (1.9)	0.02
Navitoclax	2 (1.1)	1 (1.4)	1 (0.9)	0.76
Pelabresib	1 (0.6)	1 (1.4)	0	0.39
Pacritinib	3 (1.7)	3 (4.3)	0	0.06
Venetoclax	2 (1.1)	0	2 (1.9)	0.52
Other	17 (9.7)	7 (10.1)	10 (9.4)	0.88
No treatment	28 (16.0)	9 (13.0)	19 (17.9)	0.53
Allo‐HSCT, *N* (%)	36 (21.2)	12 (17.4)	24 (23.8)	0.32
Blast percentage at baseline				
Peripheral blood, median (IQR)	11.7 (3.0–20.0)	10.0 (1.5–13.5)	15.0 (5.0–30.0)	0.001
Range	0–87	0–25	0–87	
Bone marrow, median (IQR)	20.0 (12.0–30.0)	11.8 (10.0–14.0)	25.5 (17.5–39.0)	<0.001
Range	0–90	0.5–65	0–90	

* *p*‐values refer to a comparison between the AP and BP groups using Wilcoxon's rank‐sum test for continuous variables and Fisher's exact test for discrete variables.

**FIGURE 1:**
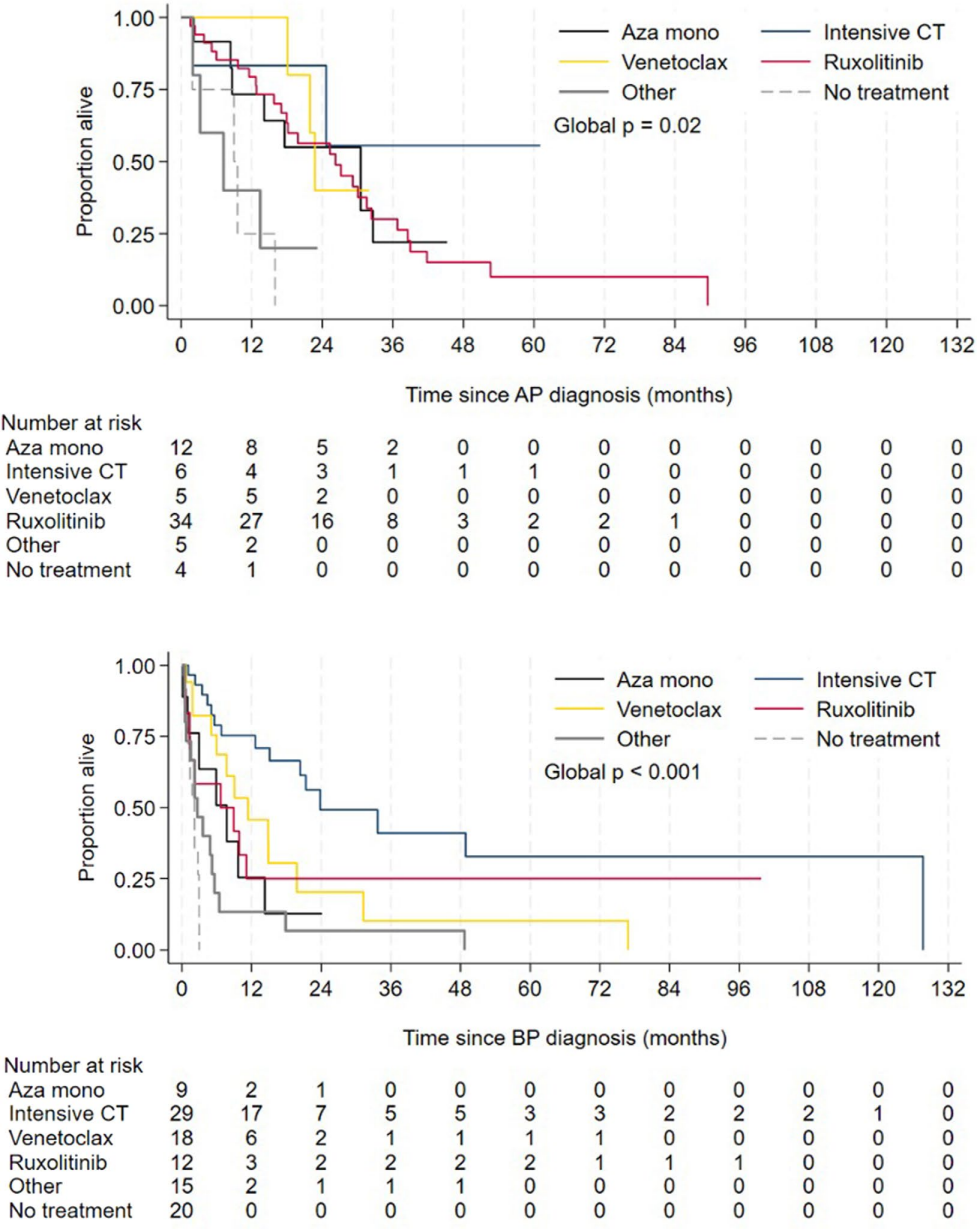
Overall survival of the entire cohort. Kaplan–Meier curves illustrating overall survival (OS) for the full study population. Patients in accelerated phase (AP) are shown in the upper panel and those in blast phase (BP) are shown in the lower panel. Survival probabilities are plotted from the time of phase diagnosis to death from any cause or last follow‐up.

**FIGURE 2:**
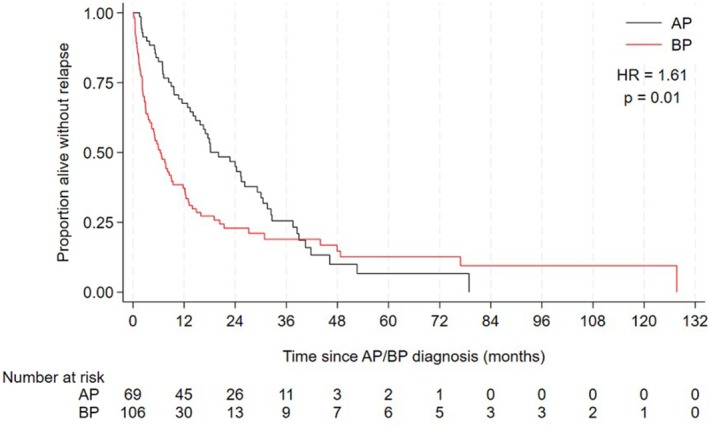
Progression‐free survival according to disease phase. Kaplan–Meier curves illustrating progression‐free survival (PFS) for patients in accelerated phase (AP) and blast phase (BP). PFS was calculated from the time of phase diagnosis to documented disease progression, relapse, transformation or death from any cause. Patients without an event were censored at the date of last follow‐up.

With a median follow‐up of 45.2 months (IQR, 17.6–79.3), median OS for the entire evaluated cohort was 14.9 months (IQR, 3.2–32.6) (Figure 1). Transformation to MPN‐BP conferred significantly inferior survival compared with MPN‐AP (median OS, 6.7 vs. 25.3 months; HR, 1.71; 95% confidence interval (CI), 1.19–2.46; *p* = 0.036). Median PFS for the entire cohort was 11.8 months (IQR, 3.2–30.5) and numerically shorter in MPN‐BP (6.4 months) than in MPN‐AP (20.1 months; HR, 1.37; 95% CI, 0.94–2.00; *p* = 0.097) (Figure 2).

There was significant heterogeneity on the treatment strategies employed in this cohort (Table 2; Table S1). In the MPN‐BP cohort, where the median age was 71.5 (IQR: 62–77) years, of interest IC was the most frequent treatment modality utilised (*n* = 29, 5 patients also had venetoclax alongside IC) followed by other venetoclax‐based regimens (*n* = 18). In the MPN‐AP cohort, where the median age was 70 (IQR: 64–75), ruxolitinib monotherapy was the most frequent treatment option chosen by clinicians (*n* = 21), followed by ruxolitinib plus azacitidine (*n* = 13) and azacitidine monotherapy (*n* = 12). A minority of patients received no treatment or only supportive care (MPN‐AP *n* = 4, MPN‐BP *n* = 20).

**TABLE 2:** Evaluable treatment responses.

Treatment group	AP			BP			Total		
	*N* = 66			*N* = 104			*N* = 170		
	*N* (%)	Median OS (IQR)	1‐year OS (95% CI)	*N* (%)	Median OS (IQR)	1‐year OS (95% CI)	Events/*N*	Median OS (IQR)	1‐year OS (95% CI)
Azacitidine monotherapy	12 (21.4%)	30.5 (8.7–32.6)	73.3% (37.9–90.6)	9 (9.4%)	7.7 (3–14.3)	25.4% (3.8–56.4)	21	14.1 (5.9–30.5)	53.5% (29.5–72.6)
Intensive chemotherapy	6 (11.8%)	‐	‐	29 (30.5%)	23.8 (12.6–127.7)	75.3% (55.1–87.4)	35	24.7 (12.6–127.7)	76.8% (58.8–87.6)
Venetoclax‐based therapies	5 (8.9%)	‐	‐	18 (18.9%)	11.4 (6–19.8)	45.8% (19.7–68.6)	23	14.9 (7.7–22.8)	59.9% (35.2–77.7)
Ruxolitinib monotherapy	21 (37.5%)	17.9 (9.7–31.6)	66.7% (42.5–82.5)	7 (7.4%)	6.7 (1.4–31)	28.6% (4.1–61.2)	28	17 (5.2–31.6)	57.1% (37.1–73)
Ruxolitinib + Aza	13 (23.2%)	36.8 (25.3–52.6)	100%	6 (6.3%)	9.9 (0.7–NA)	33.3% (4.6–67.6)	19	27.2 (12.7–52.6)	79% (53.2–91.5)
Other	5 (8.9%)	‐	‐	15 (15.8%)	2.7 (0.8–5.6)	13.3% (2.2–34.6)	20	3.2 (1.5–6.4)	20% (6.2–39.3)
No treatment/supportive treatment	4 (7.1%)	‐	‐	20 (21.1%)	2.2 (1.1–3)	‐	24	2.2 (1.3–3)	6.3% (0.4–24.8)

Regarding survival outcomes in the entire cohort of MPN = AP/BP, use of IC (median age = 60, IQR: 54–68); when followed by allo‐HSCT (median age = 59) led to the longest OS (median OS of 24.7 months (IQR 7.7–46.3)) compared to azacitidine (median age = 73, IQR: 70–77) (median OS 14.1 months (IQR: 5.9–30.5); *p* = 0.04), venetoclax‐based regimens (median age = 73.5, IQR: 64.5–75.5) (median OS of 14.9 months (IQR 3.9–32.2); *p* = 0.03) and other therapies (median age = 73, IQR: 66–80) (median OS 3.2 months (IQR: 1.5–6.4); *p* < 0.001). However, for the 11 patients who received IC but did not proceed to an allo‐HSCT, there was no significant survival difference between those and the rest of the treatment cohorts (median OS 6.8 months (IQR: 2.3–33.7) vs. 9.1 months (IQR: 2.2–27.2)).

For MPN‐AP, IC was utilised in six patients allowing them to proceed to allo‐HSCT in all cases. Ruxolitinib‐based regimens were utilised, either as monotherapy or in combination with azacitidine (*n* = 34). Ruxolitinib monotherapy achieved a median OS of 17.9 months (IQR 9.7–31.6), while the addition of azacitidine achieved a statistically significant improved median OS of 36.8 months (IQR: 25.3–52.6, *p* = 0.02 (log‐rank test)). There were no significant differences in terms of dynamic international progrnostic scoring system (DIPSS) scores and blast count between ruxolitnib monotherapy and ruxolitnib–azacitidine groups. Median OS for azacitidine alone was 30.5 months (IQR: 8.7–32.6).

For MPN‐BP, IC alone was used in 29 patients with a median OS of 23.8 (IQR: 12.6–127.7) months. Ruxolitinib‐containing regimens were used in 13 MPN‐BP patients (ruxolitinib alone *n* = 7; ruxolitinib and azacitidine *n* = 6) and performed less well with a median OS of 8.9 (IQR, 1.4–31) months and not exceeding outcomes seen with use of azacitidine alone (*n* = 9) with a median OS of 7.7 (IQR, 3–14.3) months.

Patients receiving no treatment/best supportive care had very poor outcomes, as expected, with a short median OS of 2.2 months (IQR 0.7–7.1).

**FIGURE 3:**
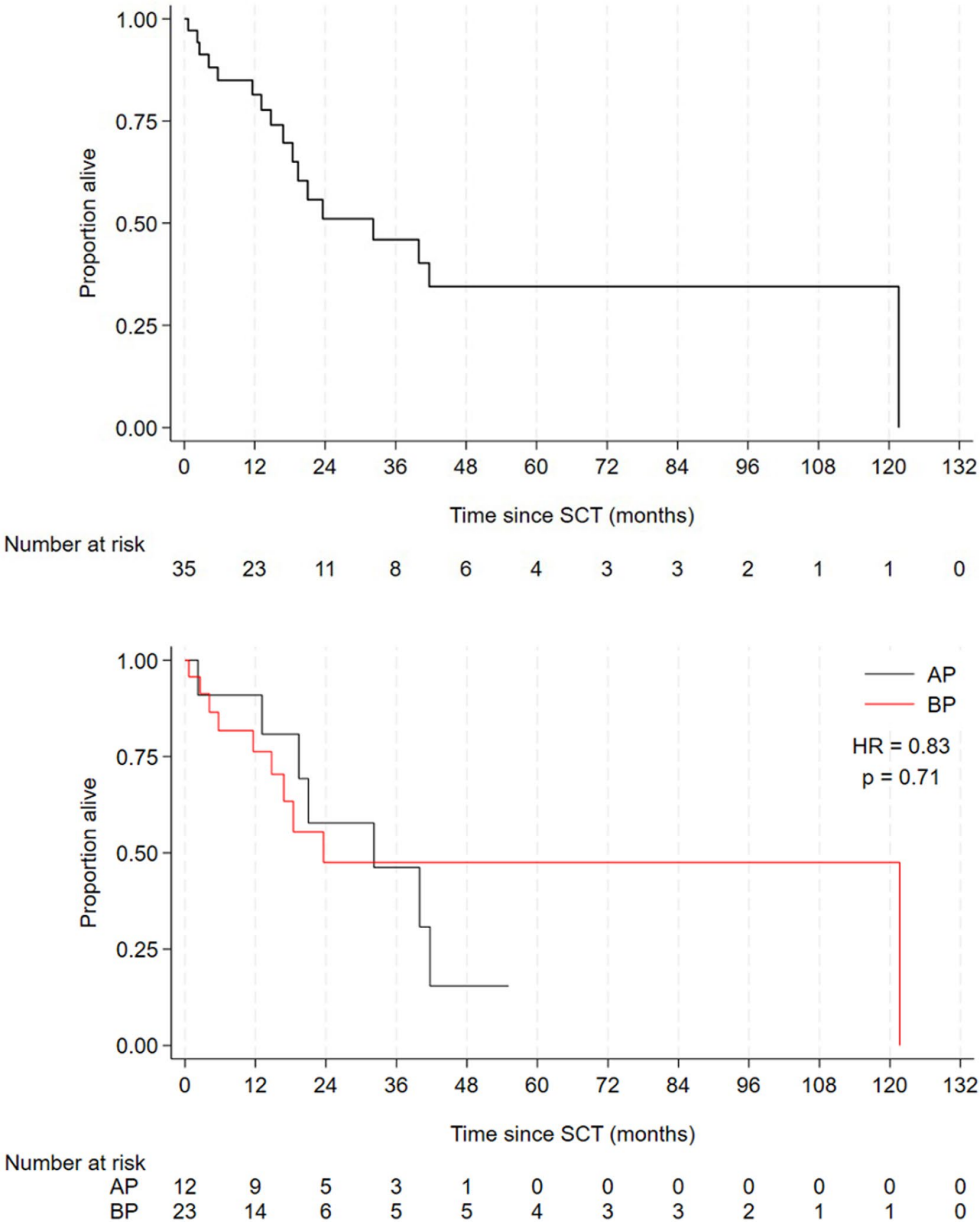
Overall survival for patients who had an allogeneic haematopoietic stem cell transplant.

Among treated patients (*n* = 151), 68 (50%) achieved an objective response. Overall response rates were highest with IC (74%) and venetoclax‐based regimens (74%). Thirty‐six patients (MPN‐BP *n* = 23 and MPN‐AP *n* = 12) (21%) proceeded to allo‐HSCT, most commonly following IC (71%; *p* < 0.001 vs. other regimens). Regarding the allo‐HSCT cohort, with a median post‐transplant follow‐up of 33.0 months, median OS and PFS were 32.2 months (IQR, 14.8–121.7) and 19.0 months (IQR, 9.0–121.7) respectively. In the AP group, median OS was 42 months (IQR: 36.8–52.6), while median PFS was 38.5 months (IQR: 20.1–52.6). In the BP group, median OS was 31 months (IQR: 20.4–127.7) (Figure 3), while median PFS was 21.4 months (IQR: 13.2–127.7) (Figure S1). The non‐relapse mortalit and relapse rate were considerable at 35.2% and 41.6%, respectively, after a median follow‐up of 33 months (IQR: 14.8–66.5) (12‐month NRM and relapse rates were 11% and 36% respectively).

Treatment‐related toxicity data were available for 155 treated patients. Grade ≥ 3 adverse events were observed in 41% overall, with clear variation as determined by regimen. Use of IC associated with the highest incidence of haematological and infectious toxicity: febrile neutropenia occurred in 49%, bacteraemia/sepsis in 20% and a pneumonia episode in 6%. Treatment‐related mortality occurred in 3%. Venetoclax‐based regimens resulted in prolonged cytopenias requiring dose interruptions in 39% of individuals and, of note, infectious complications occurred in 26%. Azacitidine monotherapy was generally well tolerated, with grade 3–4 cytopenias in 24% and grade 3–4 non‐haematological toxicity in <10%. Ruxolitinib‐containing regimens were associated with predominantly haematological toxicity (grade ≥ 3 anaemia in 18%, thrombocytopenia in 14%) but relatively low infectious risk (grade ≥ 3 bacteraemia sepsis in 17.5% and pneumonia in 14%). Other regimens (low‐dose cytarabine (*n* = 3), hydroxycarbamide (*n* = 2) or investigational agents) had low rates of grade ≥ 3 toxicity (≤10%), though efficacy was limited. Dose modification or treatment interruption for adverse events occurred in 22% of patients.

On multivariable analysis (Table 3), IC (odds ratio [OR], 10.31; 95% CI, 2.2–48.32; *p* < 0.001) and venetoclax‐based therapy (OR, 6.19; 95% CI, 1.69–22.72; *p* = 0.01) were independently associated with more favourable outcomes, after adjusting for patient age, sex, molecular profile, treatment regimen and prior therapy. When excluding patients who proceeded to transplant (Table 4), the benefit of IC showed borderline significance (OR 9.26; 95% CI 0.97–88.1; *p* = 0.05), while there was significant benefit for venetoclax‐based therapy (OR 5.83; 95% CI 1.58–21.6; *p* = 0.01) with the same covariate adjustments. In a separate univariate analyses, *TP53* (OR, 0.31; 95% CI, 0.12–0.77; *p* = 0.01) and *ASXL1* (OR, 0.60; 95% CI, 0.25–1.44; *p* = 0.26) mutations predicted inferior outcome, though only *TP53* was statistically significant. Patients presenting de novo with BP had significantly shorter OS than those with antecedent or evolving AP (HR, 1.71; *p* = 0.004).

**TABLE 3:** Multivariable analysis of factors associated with good outcome (vs. poor outcome).

Outcome	Favourable outcomes/*N*	OR	95% CI	*p*‐value
Age (years)		0.93	(0.88–0.98)	0.01
Sex				
Female	24/59	1.00	(reference)	
Male	48/104	0.57	(0.2–1.64)	0.30
Intensive chemo				
No	48/131	1.00	(reference)	
Yes	26/34	10.31	(2.2–48.32)	<0.001
Venetoclax				
No	61/144	1.00	(reference)	
Yes	13/21	6.19	(1.69–22.72)	0.01
Ruxolitinib + Aza				
No	61/147	1.00	(reference)	
Yes	13/18	11.54	(2.01–66.27)	0.01
Driver				
*JAK2*	42/103	1.00	(reference)	
*CALR*	21/35	2.40	(0.67–8.61)	0.18
*MPL* W515L	2/4	1.24	(0.03–44.49)	0.91
Triple negative	5/14	0.42	(0.06–2.9)	0.38
Prior hydroxycarbamide				
No	44/85	1.00	(reference)	
Yes	30/80	0.40	(0.14–1.11)	0.08
Prior Interferon‐Alpha				
No	49/116	1.00	(reference)	
Yes	9/17	0.56	(0.13–2.35)	0.42

**TABLE 4:** Multivariable analysis of factors associated with good outcome (vs. poor outcome) in patients who did not proceed to transplant.

Outcome	Favourable outcomes/*N*	OR	95% CI	*p*‐value
Age (years)		0.96	(0.9–1.03)	0.24
Sex				
Female	17/52	1.00	(Reference)	
Male	21/75	0.56	(0.19–1.69)	0.31
Intensive chemo				
No	37/120	1.00	(reference)	
Yes	3/9	9.26	(0.97–88.07)	0.05
Venetoclax				
No	28/109	1.00	(reference)	
Yes	12/20	5.83	(1.58–21.55)	0.01
Ruxolitinib + Aza				
No	31/115	1.00	(reference)	
Yes	9/14	12.12	(1.97–74.58)	0.01
Driver				
JAK2	26/85	1.00	(reference)	
Calreticulin	9/23	1.29	(0.28–5.93)	0.74
MPL W515L	1/3	0.91	(0.02–49.69)	0.96
Triple negative	3/12	0.54	(0.08–3.76)	0.54
Prior hydroxycarbamide				
No	20/61	1.00	(reference)	
Yes	20/68	0.48	(0.16–1.42)	0.18
Prior Interferon‐Alpha				
No	32/97	1.00	(reference)	
Yes	3/11	0.53	(0.1–2.88)	0.46

## DISCUSSION

Despite advances, management of MPN‐AP/BP remains a therapeutic challenge with very poor outcomes. This large UK‐based multi‐centre analysis of 175 patients spotlights the clinical heterogeneity of MPN‐AP/BP and a lack of a uniform treatment approach nationally. Consistent with the large US cohort by Patel et al. (*n* = 202), which reported a median OS of 10.3 months across treatment groups, our study found a relative comparable median OS of 14.9 months.^19^ Both studies underscore the poor prognosis of MPN‐BP versus AP and confirm the limited survival benefit of modern non‐transplant therapies, with no significant OS difference between IC, HMA‐based and venetoclax‐based regimens in the US analysis. Our data suggested a longer survival advantage for use of IC when followed by an allo‐HSCT, caveated by the retrospective nature of this study and the variables in clinician decision‐making for who is suitable for IC and allo‐HSCT. Venetoclax‐based therapies were also found to be associated with relatively better outcomes, but the cohort sizes are small. Regarding transplant‐ineligible patients, our findings align with the recently published French MPN study group evaluation of MPN‐AP/BP patients (*n* = 149), which reported a median OS of 8.04 months for patients treated with azacitidine alone or in combination.^20^ Notably, both our study and the French cohort analysis observed that the azacitidine–ruxolitinib combination was associated with the most favourable outcomes among non‐intensive regimens (median OS of 27.2 months in our AP cohort and 18.0 months in the French cohort). All three studies highlight the critical prognostic impact of adverse features such as occurrence of *TP53* mutations and complex karyotype and reinforce that allo‐HSCT remains the only potentially curative intervention, though clearly feasible only for a small minority.

More specifically for our cohort, treatment approaches were highly variable, reflecting the lack of a standardised approach across the United Kingdom which remains a major unmet need. However, clear efficacy differences emerged. For those patients deemed fit enough for IC (MPN‐BP = 29 and MPN‐AP = 6), they achieved the best outcomes, with a 1‐year OS of 76.8% and a median OS of 24.7 months, significantly superior to azacitidine (*p* = 0.04), venetoclax (*p* = 0.03) and ruxolitinib‐based regimens (*p* = 0.017). However, it was primarily utilised for those with MPN‐BP, achieving a median OS of 23.8 months with 25 patients proceeding to an allo‐HSCT. Venetoclax‐ (MPN‐BP = 18, MPN‐AP = 5) or azacitidine‐based therapies (MPN‐BP = 15 MPN‐AP = 25) produced only modest benefit (median OS 14.9 months), while ruxolitinib with azacitidine achieved temporary disease control (median OS: 27.2 months, 1‐year OS: 79%) but without durable remission. Notably, venetoclax‐based regimens were associated with improved outcomes on multivariable analysis after adjustment for relevant clinical and biological risk factors. Collectively, these findings suggest that, in fit patients, IC remains the most effective induction strategy, as a bridge to allo‐HSCT. The optimal role of venetoclax in this setting remains to be defined and warrants further investigation, particularly with respect to patient selection and treatment timing. Prospective studies may better identify the benefit of upfront venetoclax‐based regimens similar to the PARADIGM study in AML.^21^ In the absence of other effective treatments in the transplant‐ineligible setting, its role and efficacy should be explored further. None of our patients received IDH inhibitors reflecting that difficulty in accessing those agents in the United Kingdom. Given their success in the treatment of IDH‐mutated AML, they have been used with success in post‐MPN AML,^22^ with promising responses when used at the AP/BP phase in a short number of cases.^23^, ^24^

Toxicity profiles differed by regimen. IC was associated with high rates of febrile neutropenia (49%) and sepsis (20%), whereas venetoclax‐based regimens were complicated by prolonged cytopenias and infectious complications (26%). Ruxolitinib‐based treatments were better tolerated, as expected, but less effective overall with a median OS of 26.3 for MPN‐AP and only 8.9 months for MPN‐BP, which was not statistically superior to azacitidine monotherapy for MPN‐AP. Treatment‐related mortality, however, was low (3%), highlighting improved supportive care yet emphasising the fragility of this population.

Molecular profiling reinforced known high‐risk features across MPN‐AP/BP. *TP53* and *ASXL1* mutations independently predicted inferior outcomes (OR 0.31 and 0.60, respectively), consistent with their role in disease progression and therapeutic resistance. Of particular note, nearly one‐third of patients transformed directly from PV or ET to MPN‐AP/BP, seemingly bypassing the myelofibrosis phase. Of course, it may have been that the MF stage was not recognised prior to transformation.

Allo‐HSCT continues to represent the only potentially curative option. In this study, 21% of patients underwent allo‐HSCT, predominantly following IC (69%). Post‐transplant outcomes highlighted a median OS of 32.2 months and a 1‐year survival exceeding 70%, although relapse and infection were frequent causes of transplant failure with both high NRM (17.1%) and relapse rates (41.6%) after a median follow‐up of 33 months (IQR: 14.8–66.5). The feasibility of allo‐HSCT remains constrained, however, by advanced age, comorbidities, general frailty and the occurrence of rapid clinical deterioration typical of blastic transformation that precludes patients getting to allo‐HSCT.

Regarding limitations of the study, the retrospective design and heterogeneity in treatment selection introduce inherent biases, and molecular testing was not uniformly available across centres. Response were also assessed retrospectively by investigators at the participating centres. Nevertheless, this study represents one of the largest real‐world UK cohorts of MPN‐AP/BP patients evaluated to date. The findings emphasise that, while IC and allo‐HSCT offer the best outcomes, at least in this cohort, these remain suitable for only a minority of patients. Future studies should integrate molecularly guided therapies, potentially combining *JAK* inhibition, B‐cell Lymphoma 2 (BCL‐2) blockade and targeted agents directed at *TP53* and epigenetic modifiers, to overcome the biological resistance underlying transformation. Newer JAK inhibitors and more novel‐targeted therapies such as Chimeric Antigen Receptor T‐cell immunotherapy for *CALR*‐mutated MPNs also hold promise. Prospective international collaboration and biomarker‐driven trial design will be critical to improving outcomes in this high‐risk population.

## AUTHOR CONTRIBUTIONS

**Gabriel Naylor‐Layland:** Methodology; software; writing – review and editing; writing – original draft; data curation; formal analysis; resources; project administration. **Samah Alimam:** Writing – review and editing; resources. **Clare Brown:** Writing – review and editing; resources. **Alexandros Rampotas:** Conceptualization; methodology; formal analysis; writing – review and editing; writing – original draft; data curation; resources; project administration. **Frances Wadelin:** Writing – review and editing; resources. **Emily Booth:** Writing – review and editing; resources. **Ahmad Alabdulkarim:** Writing – review and editing; resources. **Jonathan Lambert:** Writing – review and editing; resources. **Jennifer O'Sullivan:** Writing – review and editing; resources. **Jennifer Ryan:** Writing – review and editing; resources. **Phyo Wint Wint Tun:** Writing – review and editing; resources. **Andrew J. Wilson:** Writing – review and editing; resources. **Simone Claudiani:** Writing – review and editing; resources. **Andrew Innes:** Writing – review and editing; resources. **Laith Tafesh:** Writing – review and editing; resources. **Andrew McGregor:** Writing – review and editing; resources. **Graham Greenfield:** Resources; writing – review and editing. **Mani Dubey:** Resources; writing – review and editing. **Mamta Garg:** Writing – review and editing; resources. **James Leveson:** Writing – review and editing; resources. **Wai Ka Natalie Leung:** Writing – review and editing; resources. **Steve Knapper:** Resources; writing – review and editing. **Mary Frances McMullin:** Writing – review and editing; resources. **Alesia Khan:** Resources; writing – review and editing. **Theodora Vatopoulou:** Writing – review and editing; resources. **Claire N. Harrison:** Resources; writing – review and editing. **Patrick Harrington:** Resources; writing – review and editing. **Tim C. P. Somervaille:** Writing – review and editing; resources. **Anna Godfrey:** Writing – review and editing; resources. **Bethan Psaila:** Writing – review and editing; resources. **Kate Milne:** Writing – review and editing; resources. **Amy Kirkwood:** Writing – review and editing; methodology; data curation; formal analysis. **Donal P. McLornan:** Writing – review and editing; project administration; resources; supervision; writing – original draft; conceptualization; methodology; investigation. **Charlotte Brierley:** Resources; writing – review and editing. **Duncan Brian:** Resources; writing – review and editing.

## CONFLICT OF INTEREST STATEMENT

CH: Advisory/Consultancy roles: AbbVie, AOP, BMS/Celgene, CTI BioPharma, Galecto, Geron, GSK, IMAGO, Incyte, Ionis, Janssen, Keros, MSD, MorphoSys/Constellation, Novartis, Silence, Sobi. Research funding (institutional/PI): AbbVie, GSK, MorphoSys/Constellation, Novartis. Honoraria/Speakers Bureau: AbbVie, AOP, BMS, IMAGO, Incyte, Keros, MSD, Novartis. DPM: Research Funding: Imago Biosciences, Honoraria: AbbVie, Jazz Pharmaceuticals, Novartis. TS: Consultancy: BMS, GSK, MSD, Novartis. Research Funding: CellCentric Ltd. BP: Consultancy/Advisory board: BMS, Blueprint Medicines, Incyte, Novartis. Research Funding: Incyte. Honoraria: GSK, Novartis. PH: Honoraria and Research Funding: AOP, Constellation, GSK, Incyte, Novartis. JL: Consultancy/Honoraria: Blueprint Medicines, Kite‐Gilead, Novartis, Takeda. AG: Advisory board/Membership: AOP, Celgene/BMS, Novartis, Honoraria/Speakers Bureau: Novartis. AI: Advisory board/Speakers Bureau: Novartis, Speakers Bureau: Incyte, Other: Conference fees: Novartis. AM: Advisory board/Speakers Bureau/Conference fees: Novartis. Speakers Bureau: GSK. FW: Advisory board: GSK, Speakers Bureau: BMS, Novartis. MFM: Advisory board: BMS, GSK, Novartis, Speakers Bureau: GSK, Incyte, Novartis. Clinical trial support: BMS. SC: Honoraria: Incyte, Pfizer. The rest of the authors declare no relevant conflict of interest.

## Supporting information


Figure S1.

Table S1.

## Data Availability

The data that support the findings of this study are available on request from the corresponding author. The data are not publicly available due to privacy or ethical restrictions.
