# Supplementary figures and images for: Real‐world treatment patterns and outcomes in accelerated and blast‐phase myeloproliferative neoplasms: Insights from a large multi‐centre cohort analysis in the United Kingdom
Source: Br J Haematol. 2026 May 3;209(1):160–71. doi: 10.1111/bjh.70511 (PMC13340518; doi:10.1111/bjh.70511)

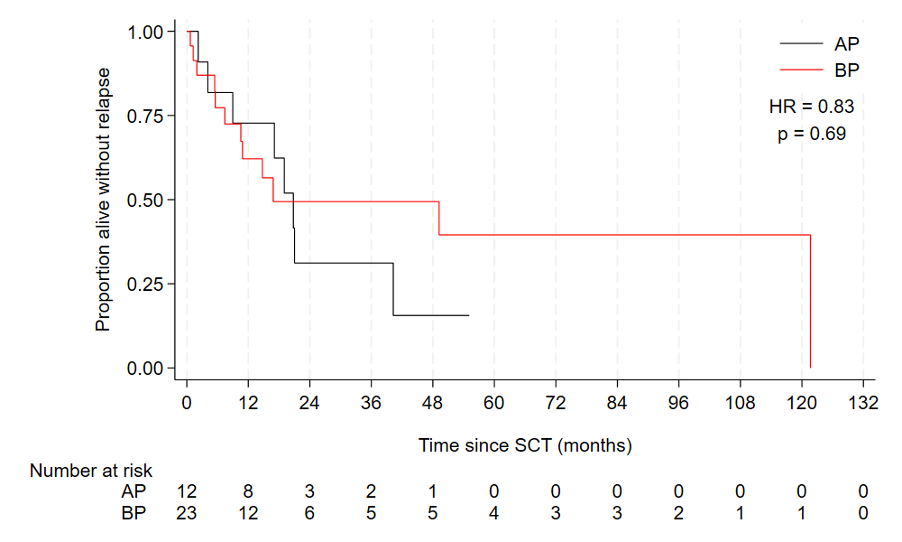

Supplement: Supplementary file 1 — Figure S1. [file BJH-209-160-s001.png]
