# Supplementary material for: Real‐world treatment patterns and outcomes in accelerated and blast‐phase myeloproliferative neoplasms: Insights from a large multi‐centre cohort analysis in the United Kingdom
Source: Br J Haematol. 2026 May 3;209(1):160–71. doi: 10.1111/bjh.70511 (PMC13340518; doi:10.1111/bjh.70511)
Supplement: Supplementary file 2 — Table S1. [file BJH-209-160-s002.docx]

**Table S1 – Treatment categorisation**

| **Treatment** | **Treatment group** | **Number of patients** |
| --- | --- | --- |
| Azacitidine | Azacitidine monotherapy | 21 |
| DA | Intensive chemo | 5 |
| DA+ FLAGIDA_FLA_IDA | Intensive chemo | 2 |
| DA+ FLAGIDA_FLA_IDA+ Venetoclax+ Azacitidine | Intensive chemo | 1 |
| DA+ FLAGIDA_FLA_IDA+ Venetoclax+ Other (Gemtuzumab ozogamicin) | Intensive chemo | 1 |
| DA+ Other (Gemtuzumab ozogamicin) | Intensive chemo | 1 |
| DA+ Other (HiDAC/HDAC) | Intensive chemo | 1 |
| DA+ Venetoclax+ Azacitidine | Intensive chemo | 1 |
| FLAGIDA_FLA_IDA | Intensive chemo | 8 |
| FLAGIDA_FLA_IDA+ Azacitidine+ Other (DLI) | Intensive chemo | 1 |
| FLAGIDA_FLA_IDA+ VYXEOS | Intensive chemo | 3 |
| FLAGIDA_FLA_IDA+ VYXEOS+ Venetoclax+ Azacitidine | Intensive chemo | 1 |
| FLAGIDA_FLA_IDA+ VYXEOS+ Venetoclax+ Ruxolitinib+ Azacitidine+ Other (HiDAC/HDAC) | Intensive chemo | 1 |
| FLAGIDA_FLA_IDA+ Venetoclax+ Azacitidine | Intensive chemo | 1 |
| FLAGIDA_FLA_IDA+ Venetoclax+ Other (CPX) | Intensive chemo | 1 |
| Other(CPX) | Intensive chemo | 1 |
| VYXEOS | Intensive chemo | 5 |
| VYXEOS+ Venetoclax+ Azacitidine | Intensive chemo | 1 |
| Venetoclax+ Azacitidine | Venetoclax | 18 |
| Venetoclax+ Azacitidine+ Notreatmentreceived | Venetoclax | 1 |
| Venetoclax+ Azacitidine+ Other (Anagrelide) | Venetoclax | 1 |
| Venetoclax+ Azacitidine+ Other (Cytarabine) | Venetoclax | 1 |
| Venetoclax+ Azacitidine+ Other (Hydroxycarbamide) | Venetoclax | 1 |
| Venetoclax+ LDAC+ Ruxolitinib+ Azacitidine | Venetoclax | 1 |
| Ruxolitinib | Ruxolitinib mono | 16 |
| Ruxolitinib+ Momemolitinib | Ruxolitinib mono | 1 |
| Ruxolitinib+ Navitoclax | Ruxolitinib mono | 1 |
| Ruxolitinib+ Other (Darbopoietin) | Ruxolitinib mono | 2 |
| Ruxolitinib+ Other (EPO) | Ruxolitinib mono | 1 |
| Ruxolitinib+ Other (Hydroxycarbamide) | Ruxolitinib mono | 6 |
| Ruxolitinib+ Other (Prednisolone) | Ruxolitinib mono | 1 |
| Ruxolitinib+ Azacitidine | Ruxolitinib + Aza | 16 |
| Ruxolitinib+ Azacitidine+ Other (Cytarabine) | Ruxolitinib + Aza | 1 |
| Ruxolitinib+ Azacitidine+ Other (EPO) | Ruxolitinib + Aza | 1 |
| Ruxolitinib+ Azacitidine+ Other (Hydroxycarbamide) | Ruxolitinib + Aza | 1 |
| Fedratinib+ Other (Hydroxycarbamide) | Other | 1 |
| Low dose cytarabine (LDAC) | Other | 3 |
| Momelotinib | Other | 1 |
| Other (Cytarabine) | Other | 1 |
| Other (Hydroxycarbamide and Cytarabine) | Other | 1 |
| Other (Hydroxycarbamide and EPO) | Other | 1 |
| Other (Hydroxycarbamide) | Other | 10 |
| Other (Inobrodib) | Other | 1 |
| Other (Prednisolone) | Other | 1 |
| No treatment received | No treatment | 24 |

DA = Daunorubicin + Cytarabine, FLAG-IDA / FLA-IDA / FLAGIDA_FLA_IDA = Fludarabine + Cytarabine + Granulocyte Colony-Stimulating Factor (G-CSF) + Idarubicin, HiDAC / HDAC = High-Dose Cytarabine, LDAC / LD Cytarabine = Low-Dose Cytarabine, VYXEOS / CPX = CPX-351 (Liposomal Daunorubicin + Cytarabine), EPO = Erythropoietin, DLI = Donor Lymphocyte Infusion, CCS-1477 (Inobrodib) = Bromodomain and Extra-Terminal (BET) Protein Inhibitor.
